# Diagnostic accuracy of cerebrospinal fluid protein markers for sporadic Creutzfeldt-Jakob disease in Canada: a 6-year prospective study

**DOI:** 10.1186/1471-2377-11-133

**Published:** 2011-10-27

**Authors:** Michael B Coulthart, Gerard H Jansen, Elina Olsen, Deborah L Godal, Tim Connolly, Bernard CK Choi, Zheng Wang, Neil R Cashman

**Affiliations:** 1 Canadian Creutzfeldt-Jakob Disease Surveillance System, Public Health Agency of Canada, 1015 Arlington Street, Winnipeg MB R3E 3R2, Canada; 2 Canadian Creutzfeldt-Jakob Disease Surveillance System, Public Health Agency of Canada, 200 Églantine Driveway AL 1910B, Ottawa ON K1A 0K9, Canada; 3 Department of Pathology and Laboratory Medicine, Eastern Ontario Regional Laboratory, CCW 4240B, The Ottawa Hospital - General Campus, 501 Smyth Rd, Ottawa ON K1H 8L6, Canada; 4 Chronic Disease Surveillance and Monitoring Division, CCDPC, HPCDPB, Public Health Agency of Canada, Room 622A3, 785 Carling Avenue, PL# 6806A, Ottawa ON K1A 0K9, Canada; 5 Department of Epidemiology and Community Medicine, University of Ottawa, ON, Canada; 6 Shantou University Medical College, Shantou, China; 7 Brain Research Centre and PrioNet Canada, University of British Columbia, 2011 Wesbrook Mall, Vancouver BC V6T 2B5, Canada

## Abstract

**Background:**

To better characterize the value of cerebrospinal fluid (CSF) proteins as diagnostic markers in a clinical population of subacute encephalopathy patients with relatively low prevalence of sporadic Creutzfeldt-Jakob disease (sCJD), we studied the diagnostic accuracies of several such markers (14-3-3, tau and S100B) in 1000 prospectively and sequentially recruited Canadian patients with clinically suspected sCJD.

**Methods:**

The study included 127 patients with autopsy-confirmed sCJD (prevalence = 12.7%) and 873 with probable non-CJD diagnoses. Standard statistical measures of diagnostic accuracy were employed, including sensitivity (Se), specificity (Sp), predictive values (PVs), likelihood ratios (LRs), and Receiver Operating Characteristic (ROC) analysis.

**Results:**

At optimal cutoff thresholds (empirically selected for 14-3-3, assayed by immunoblot; 976 pg/mL for tau and 2.5 ng/mL for S100B, both assayed by ELISA), Se and Sp respectively were 0.88 (95% CI, 0.81-0.93) and 0.72 (0.69-0.75) for 14-3-3; 0.91 (0.84-0.95) and 0.88 (0.85-0.90) for tau; and 0.87 (0.80-0.92) and 0.87 (0.84-0.89) for S100B. The observed differences in Sp between 14-3-3 and either of the other 2 markers were statistically significant. Positive LRs were 3.1 (2.8-3.6) for 14-3-3; 7.4 (6.9-7.8) for tau; and 6.6 (6.1-7.1) for S100B. Negative LRs were 0.16 (0.10-0.26) for 14-3-3; 0.10 (0.06-0.20) for tau; and 0.15 (0.09-0.20) for S100B. Estimates of areas under ROC curves were 0.947 (0.931-0.961) for tau and 0.908 (0.888-0.926) for S100B. Use of interval LRs (iLRs) significantly enhanced accuracy for patient subsets [*e.g*., 41/120 (34.2%) of tested sCJD patients displayed tau levels > 10,000 pg/mL, with an iLR of 56.4 (22.8-140.0)], as did combining tau and S100B [*e.g*., for tau > 976 pg/mL and S100B > 2.5 ng/mL, positive LR = 18.0 (12.9-25.0) and negative LR = 0.02 (0.01-0.09)].

**Conclusions:**

CSF 14-3-3, tau and S100B proteins are useful diagnostic markers of sCJD even in a low-prevalence clinical population. CSF tau showed better overall diagnostic accuracy than 14-3-3 or S100B. Reporting of quantitative assay results and combining tau with S100B could enhance case definitions used in diagnosis and surveillance of sCJD.

## Background

Prion diseases are rare neurodegenerative disorders that arise sporadically, genetically, or by infectious transmission. They are marked by spongiform change, neuronal loss, and deposition of a misfolded host-encoded glycoprotein (PrP^Sc^) in brain tissue that is generally considered to constitute the transmissible agent [1]. Despite their rarity, the economic and public-health impacts of human prion diseases [2] maintain a need for detailed surveillance and prompt diagnosis, usually with support from expert reference units [3]. Sporadic Creutzfeldt-Jakob disease (sCJD) accounts for 80-90% of annual human prion disease mortality (~1-2 per million) [4]. With its subtype heterogeneity [5] and diverse presenting symptoms [6] that may accompany other conditions [7], differentiation of sCJD from other subacute encephalopathies can challenge the clinician, especially on a first encounter [8]. Surveillance-oriented sCJD case definitions are available [3], with recent updates including MRI criteria [9]. However, definitive, non-invasive diagnostic laboratory tests for sCJD that could be applied to the living patient remain elusive, largely because pathologic PrP^Sc ^is found only in trace amounts in body fluids. Recent technical developments promise to eventually allow the reliable ultrasensitive detection of PrP^Sc ^in such samples [10,11]; however, for the moment definitive diagnosis of sCJD remains highly dependent on direct examination of brain tissue [12].

Certain brain proteins found in cerebrospinal fluid (CSF) - in particular 14-3-3 proteins, microtubule-associated protein tau, neuron-specific enolase, and S100B - have demonstrated their utility as sCJD markers [13-15] but produce some discordant results that have raised questions regarding their fitness for diagnostic purposes [16,17]. In one large multi-center study for example [13], diagnostic sensitivity and specificity for sCJD were estimated at 0.85 and 0.84 respectively for CSF 14-3-3 protein; 0.86 and 0.88 for microtubule-associated protein tau; 0.73 and 0.95 for neuron-specific enolase; and 0.82 and 0.76 for S100B. Some single-centre studies have reported better performance, although still with false positives and false negatives [14,15].

Such inaccuracies can have significant negative impact on patients and institutions, prompting advice that 14-3-3 in particular should only be used within an "appropriate clinical context" [18], or perhaps should not be used [17]. Nevertheless, surveillance case definitions of probable sCJD still include a positive 14-3-3 assay [3], and use of 14-3-3 reference services is positively correlated with observed sCJD mortality rates [19]. The use of CSF markers in sCJD diagnosis *per se *is also likely to continue - the 14-3-3 assay in particular remains the most frequently requested laboratory test for human prion diseases. Precise quantitative estimates of these markers' performance characteristics are therefore needed, for timely revision of diagnostic probabilities [20,21]. Such a framework should also be applicable to screening of a broad range of patients for whom a suspicion of sCJD exists, and not only to strengthen confirmation of the disease (or its absence) in a select group for whom pre-test diagnostic probabilities are already highly refined [22].

The Public Health Agency of Canada's Creutzfeldt-Jakob Disease Surveillance System (CJDSS) has conducted prospective, autopsy-based surveillance of human prion diseases in Canada since April 1 1998, with the objective of identifying and characterizing all cases of human prion disease in Canada. As final diagnostic classifications and most laboratory tests (including all CSF testing) are performed centrally using standard methods and criteria [3], we were afforded an opportunity to simultaneously validate the respective abilities of several known CSF protein markers (14-3-3, tau and S100B) to differentiate sCJD from a broad range of other conditions prompting suspicion of sCJD. Here we report results of a prospective study of diagnostic accuracies of CSF 14-3-3, tau and S100B proteins in 1000 sequentially recruited Canadian patients, including 127 with neuropathologically confirmed sCJD. Our results indicate that these markers can have substantial diagnostic power even when used in a heterogeneous patient population with low average pre-test probability of sCJD. A preliminary abstract of these findings has been previously published [23].

## Methods

### Participants

CJD surveillance takes place in Canada through direct case-by-case consultation between the CJDSS and collaborating health professionals, with laboratory reference services provided by the CJDSS for neuropathology, molecular genetics, and CSF 14-3-3 protein testing. Following initial identification of a suspected case, a standard intake questionnaire is completed to collect information on the patient's symptoms, signs, history, results of previous investigations, and current status. The case is then followed throughout the course of the patient's illness, as long as prion disease remains suspected and the prospect of patient enrolment for full case investigation remains open. Case files are maintained centrally and remain open until all available information has been collected. Infectious and genetic risk factor information is investigated by a standardized questionnaire and medical chart review; cases are then classified using internationally standardized criteria [3]. Non-prion disease (non-CJD) diagnoses are routinely ascertained wherever possible, either directly *via *CJDSS laboratory investigations, or by elicitation from the collaborating health professional at the time of file closure.

### Laboratory procedures

Formalin-fixed brain tissues obtained by autopsy or biopsy were processed for neuropathological examination using standard techniques. Slides were stained with haematoxylin and eosin, Periodic Acid-Schiff's reagent, and anti-prion protein immunohistochemistry with mouse monoclonal antibody 12F10 (189710, Cayman Chemical, Ann Arbor, MI, USA) at a 1:2000 working dilution. Additional stains were used when an alternative diagnosis was suspected based on histological findings. Slides were evaluated by two expert reviewers [3].

Codons 3-254 and 101 nucleotides of the 3' untranslated region of exon 2 in the *PRNP *gene were amplified by polymerase chain reaction with primers D (5' GCAGAGCAGTCATTATGG 3') and E (5' CCTCAACCTGTTGCACTAAGTCC 3'), and sequenced on both strands with multiple oligodeoxynucleotide primers using dideoxynucleotide chain-termination chemistry.

CSF specimens were shipped to the CJDSS laboratory on dry ice, thawed upon receipt, and either analyzed on the same day or immediately re-frozen and stored at -80°C pending analysis. 14-3-3 protein was assayed using a slightly modified version of a standard chemiluminescence-based immunoblotting protocol [18] on a 20-μL aliquot of CSF. Immunodetection employed a mouse monoclonal primary antibody against the N-terminus of human 14-3-3 β (SC-1657, Santa Cruz Biotechnology, Santa Cruz, CA, USA). Each gel included internal standards, consisting of a standard dilution series of recombinant human 14-3-3 γ protein (National Institute for Biological Standards and Control, Potters Bar, Hertsfordshire, UK; or prepared in-house - note that SC-1657 cross-reacts with human 14-3-3 γ). The positive/negative scoring threshold for 14-3-3 was initially selected to consistently produce a faint band on the X-ray film, and corresponded to the immunoreactivity of approximately 1.5 ng of recombinant 14-3-3 γ protein per lane. Films were scored by a single observer (MBC) throughout, with concurrence from a second observer (DLG).

After reporting of the 14-3-3 result, where sufficient sample remained tau and S100B protein assays were performed on the same CSF specimens, ensuring a fully prospective, sequential, "one-gate" design blinded to the final diagnostic result. Assays for CSF tau and S100B proteins were performed using Innotest^® ^hTAU Ag (Innogenetics, Ghent, Belgium) and Sangtec^® ^100 (Diasorin, Saluggia, Italy) ELISA kits, following instructions provided by the manufacturer. Interim storage of samples between analytic procedures was at -80°C, and freeze-thaw cycles were kept to a minimum after receipt from submitting laboratories. Calibration standards for ELISA assays were reconstituted fresh for each run. Lot-specific internal controls with expected ranges of values are provided by the manufacturer of the Sangtec^® ^100 kit, and were run with every assay; such control materials are not yet available for the Innotest^® ^hTAU Ag kit. In all cases 2 replicate assays were carried out on each clinical sample, control or calibration standard, and the arithmetic mean of the 2 readings was taken as the sample result. The criterion for acceptance of an assay result (which was not violated in the course of this study) was that the coefficient of variation of the 2 replicates within a run was no greater than 20%. Sample readings outside the ranges of kit standards were resolved if possible by sample dilution using diluents provided with the kit. Where this was not possible, for purposes of data analysis assay results were equated with concentrations of the uppermost or lowermost standards.

### Composition and statistical analysis of dataset

CSF marker data were collected prospectively from the ongoing series of samples submitted to the CJDSS for 14-3-3 protein testing. Data were analyzed from the first 1000 contiguous CSF samples submitted after April 1, 2004 for which 14-3-3 tests were completed, final patient diagnoses were available, and none of the following exclusion criteria applied: (i) the sample was technically inadequate for 14-3-3 testing (insufficient volume, xanthochromia or visible blood); (ii) more than one technically adequate sample was submitted for the same patient (results from one of the replicate samples were then randomly included for analysis); (iii) the CJDSS was unable to confirm that prion disease was suspected at the time of CSF sample submission (clinical or clerical errors); (iv) the 14-3-3 assay result was indeterminate due to molecular-weight anomalies of reactive bands on the immunoblot; (v) final diagnostic classification was genetic prion disease; (vi) final diagnostic classification was probable sCJD (as a positive 14-3-3 result was used as a criterion to classify such cases, they could not be included in the validation study for this marker); or (vii) the case remained open at study closure (July 31 2010). For 946 of these 1000 samples sufficient volume was available to assay total tau protein, and for 924 to assay S100B protein.

Statistical and graphical procedures were executed with MedCalc^® ^for Windows v. 11.2.1.0 (MedCalc Software, Mariakerke, Belgium), Confidence Interval Analysis [24], Post_Test_Probabilities [25], and online clinical research calculators available on the Vassar Stats website [26]. Relevant definitions and theory are presented by Sackett *et al*. [20] and Grimes [21]. Diagnostic cutoff thresholds are denoted in the text by subscripts; *e.g*., T_976 _for 976 pg/mL of tau, and S_2.5 _for 2.5 ng/mL of S100B. To determine whether our study sample was of adequate size to perform valid logistic regression analyses, an estimate of the minimum number of cases required is given by N = 10 k/p, where k is the number of independent variables, and p is the smaller of the proportions of positive and negative cases in the population sample [27]. We performed two logistic regression analyses with k = 2 or 3, and p = 0.127 (for sCJD) in both cases; thus, N = 157.5 or 236.2. Since the number of cases in each of these analyses was 913, this criterion was satisfied.

### Ethics statement

Enrolment in the CJDSS takes place with written informed consent, under a protocol approved by the Health Canada - Public Health Agency of Canada Research Ethics Board (Certificate REB-2009-0036).

## Results

### Study population

**Figure 1:**
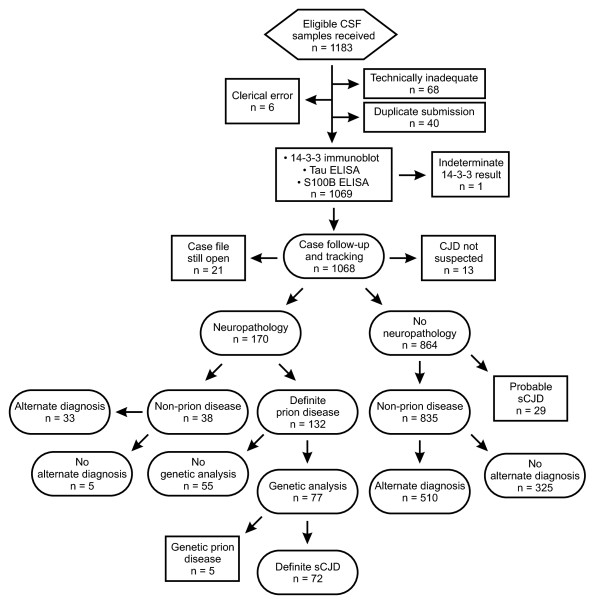
**Sample intake, laboratory testing, case follow-up and final case classification**. Numbers of CSF samples received and processed, and numbers of suspected CJD cases followed up, examined by neuropathology and classified, used to constitute the clinical population on which the study was based. Details of inclusion and classification criteria are explained further in the text.

Between April 1 2004 and July 31 2010, a total of 1,183 CSF samples were received by the CJDSS reference laboratory for 14-3-3 testing. Of the 1000 of these selected for inclusion in the study (see Methods), in 127 cases sCJD was subsequently confirmed by positive neuropathology without evidence of an infectious or genetic cause. Of the 873 non-CJD cases, 543 (62.2%) were assigned a specific final diagnosis other than prion disease (see Additional file: 1, Table S1), and 330 (37.8%) had an unknown diagnosis but no further suspicion of prion disease, thereby qualifying them as "probable non-CJD". A total of 29 patients with a diagnosis of probable sCJD were excluded from the statistical analysis (see Methods). No final diagnoses of possible sCJD were assigned. Minimum prevalence of sCJD in the study population was thus estimated to be 127/1000, or 0.127 [95% Confidence Interval (CI): 0.107-0.150]. A flowchart summarizing numbers of samples, tests and diagnoses is shown in Figure 1.

Median age of sCJD cases at notification was 66 (95% CI: 63-69) years, and that of the 866 non-CJD cases for whom data were available was 63 (95% CI: 62-64) years. Numbers of *PRNP *codon 129 genotypes among the 72 sCJD patients tested were 44 MM (61.1%), 16 MV (22.2%), and 12 VV (16.7%). Of the sCJD cases, 66/127 (52.0%) were male; of non-CJD cases, 448/873 (51.3%).

### Single marker performance

**Figure 2:**
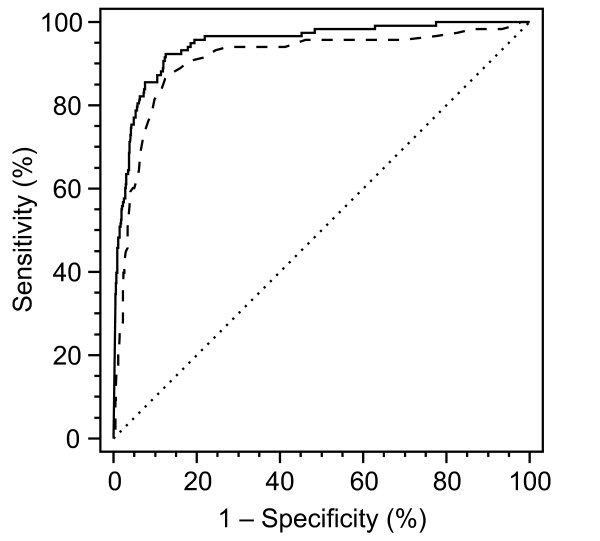
**Receiver Operating Characteristic (ROC) curves for tau and S100B, plotted for 913 sCJD and non-CJD patients on whom both markers were assayed**. Solid line indicates ROC curve for tau; dashed line indicates ROC curve for S100B; dotted line indicates diagonal representing a hypothetical test with no diagnostic discrimination.

Receiver operating characteristic (ROC) curves were plotted for tau and S100B in the 913 cases tested for both markers (Figure 2). Diagnostic sensitivity (Se) and specificity (Sp) were co-optimized by maximizing the Youden Index (Se + Sp - 1) at cutoff thresholds of 976 pg/mL (T_976_) for tau and 2.5 ng/mL (S_2.5_) for S100B. Estimates of area under the curve (AUC) were 0.95 (95% CI: 0.93-0.96) for tau and 0.91 (95% CI: 0.89-0.93) for S100B, implying significant discriminatory power of both markers in this patient population (P < 0.0001 for AUC > 0.5, DeLong test) as well as slight but statistically significant differences between them (P = 0.03, DeLong test).

Se, Sp, and positive and negative likelihood ratios (LR^+^, LR^-^) were estimated for 14-3-3, tau and S100B (Table 1), applying either our estimated optimal thresholds or, for the ELISA assays, consensus thresholds (T_1300_, S_4.2_) widely applied by other CJD reference laboratories using the same reagent kits [13]. With optimal thresholds, five of the six Se and Sp estimates were in the range 0.84-0.91, with the exception being 14-3-3 Sp at 0.72 (95% CI: 0.69-0.75). Newcombe's method for differences of proportions [24] was used to test all 6 pairwise differences of Se and Sp between markers. Based on non-inclusion of zero in 95% confidence intervals for these differences the only significant differences occurred between Sp of 14-3-3 and that of either of the other two markers; this comparison was significant regardless of whether all non-CJD cases were included, or only those with specific non-CJD diagnoses. As expected, use of the higher consensus thresholds decreased Se and increased Sp for both tau and S100B.

**Table 1:** Performance characteristics for 14-3-3, tau and S100B.

Marker	Threshold	**Se**^**a**^	95%CI	**Sp**^**a**^	95% CI	**LR**^**+ a**^	95% CI	**LR**^**-a**^	95% CI
14-3-3	-	0.88	0.81-0.93	0.72	0.69-0.75	3.1	2.8-3.6	0.16	0.10-0.26
Tau	976^b^	0.91	0.84-0.95	0.88	0.85-0.90	7.4	6.9-7.8	0.10	0.06-0.20
	1300^c^	0.84	0.76-0.90	0.92	0.90-0.94	10.9	8.5-13.9	0.17	0.11-0.26
S100B	2.5^b^	0.87	0.80-0.92	0.87	0.84-0.91	6.6	6.1-7.1	0.15	0.09-0.20
	4.2^c^	0.52	0.42-0.61	0.97	0.95-0.98	15.3	10.2-23.1	0.50	0.42-0.60

^a ^Se - sensitivity, Sp - specificity, LR^+ ^- positive likelihood ratio, LR^- ^- negative likelihood ratio.

^b ^Optimal scoring thresholds for tau and S100B, estimated in this study. Values are in pg/mL for tau, and in ng/mL for S100B.

^c ^Consensus scoring thresholds for tau and S100B, based on published literature.

LR^+ ^estimates at optimal thresholds were lowest for 14-3-3 at 3.1 (95% CI: 2.8-3.6), *versus *those for tau and S100B at 7.4 (95% CI: 6.9-7.8) and 6.6 (95% CI: 6.1-7.1), respectively. LR^- ^estimates were lowest for tau at 0.10 (95% CI: 0.06-0.20), *versus *those for 14-3-3 and S100B at 0.16 (95% CI: 0.10-0.26) and 0.15 (95% CI: 0.09-0.20), respectively. Again, the estimates for tau and S100B all shifted when the consensus thresholds were applied (Table 1). We also calculated interval likelihood ratios (iLRs) for illustrative sub-ranges of tau and S100B assay results (Table 2). Significant variation was observed across intervals in their power to support or exclude a diagnosis of sCJD. With tau for example, above T_10,000 _the iLR rose significantly to 56.4 (95% CI: 22.8-140.0), and below T_500 _fell to 0.06 (95% CI: 0.03-0.14). With S100B, above S_4.0 _the iLR was 14.6 (95% CI: 10.0-21.4), and below S_2.0 _was 0.09 (95% CI: 0.05-0.18).

**Table 2:** Interval likelihood ratios for tau and S100B

Marker	**Interval**^**a**^	**N (sCJD)**^**b**^	**N (non-CJD)**^**b**^	**iLR**^**c**^	95% CI
Tau	0-500	5	591	0.06	0.03-0.14
	500-1,000	10	138	0.50	0.27-0.92
	1,000-3,000	24	65	2.5	1.7-3.9
	3,000-10,000	40	27	10.2	6.5-16.0
	10,000-∞	41	5	56.4	22.8-140.0
S100B	0-2.0	8	581	0.09	0.05-0.18
	2.0-3.0	19	147	0.85	0.55-1.3
	3.0-4.0	26	43	4.0	2.5-6.2
	4.0-**∞**	69	31	14.6	10.0-21.4

^a ^Interval values are in pg/mL for tau, and in ng/mL for S100B.

^b ^Numbers of patients with test results falling into each interval are shown.

^c ^iLR - interval likelihood ratio. Values are estimates of iLR for CSF tau and S100B at illustrative cutoff thresholds.

### Joint marker performance

**Figure 3:**
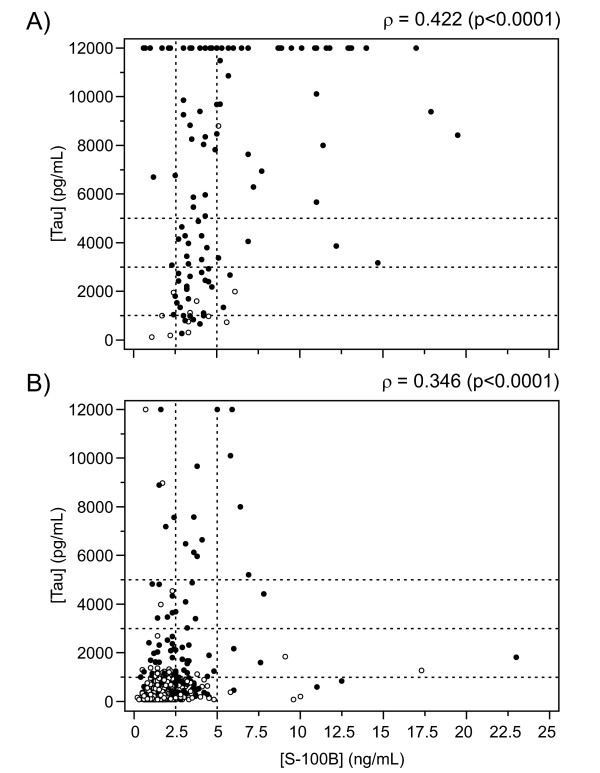
**CSF tau, S100B, and 14-3-3 joint distributions**. Scatter plots of CSF concentrations of tau and S100B for (A) 118 sCJD cases and (B) 795 non-CJD cases. Dotted lines mark thresholds of 1,000, 3,000 and 5,000 pg/mL for tau, and 2.5 and 5.0 ng/mL for S100B. Filled and open circles indicate cases with positive and negative 14-3-3 results, respectively. To maintain S100B scales commensurate between plots, two non-CJD cases with S100B levels of 43.7 and 46.5 ng/mL and tau levels of 755.4 and 2,182 pg/mL, respectively, were excluded from plot B. Values of Spearman's ρ and respective significance levels are provided for each scatter plot.

Tau and S100B values were plotted jointly for 118 sCJD and 795 non-CJD cases tested for both markers (Figure 3). The two markers were significantly correlated [Spearman's ρ, 0.422 (95% CI: 0.261-0.560, p < 0.0001) for sCJD; and 0.346 (95% CI: 0.283-0.406, p < 0.0001) for non-CJD]. However, sCJD cases showed a visible tendency toward simultaneous elevation of both markers, while a large majority of non-CJD data points fell closer to one or both axes.

To quantitatively assess the potential added diagnostic power of combining tau and S100B results *in this study population only*, we performed a multiple logistic regression analysis with tau and S100B as independent variables and logit of diagnosis (sCJD) as the dependent variable. The overall regression model, logit(p) = -3.4907 + 0.0005279 × [tau] + 0.09543 × [S100B] was statistically significant (P < 0.0001, Chi-square likelihood ratio test, 2 df). P values were < 0.0001 and 0.0052 for the regression coefficients of [tau] and [S100B] respectively, supporting the conclusion that each marker had a significant positive effect on the probability of a diagnosis of sCJD. ROC curves for tau, S100B and their joint regression-derived bivariate risk scores were then compared. As mentioned earlier, AUC estimates for the subset of 913 patients who were tested for both markers were 0.95 (95% CI: 0.93-0.96) for tau and 0.91 (95% CI: 0.89-0.93) for S100B; AUC for the bivariate risk score was 0.96 (95% CI: 0.94-0.97). Pairwise comparisons of AUC between both bivariate risk score *versus *tau, and bivariate risk score *versus *S100B, showed significant differences (P = 0.0241 and 0.0051, respectively), supporting the interpretation that combining these two markers increased overall discriminatory power over that of either marker alone in our study population.

To further characterize the joint diagnostic performance of tau and S100B using a different approach, we constructed 2 × 2 contingency tables using 6 illustrative discrete bivariate cutoff thresholds defined by combinations of (S_2.5 _OR S_5.0_) AND (T_976 _OR T_3000 _OR T_5000_). Resulting joint LR^+ ^estimates (Table 3) ranged from 18.2 (95% CI: 12.9-25.7) for S_2.5 _+ T_976_, to 57.3 (95% CI: 20.7-158.5) for S_5.0 _+ T_5000_. Thus, in our study population a combination of S100B > 5.0 ng/mL and tau > 5000 pg/mL affords as much power to rule in sCJD as a tau result of > 10,000 pg/mL, with its individual iLR estimate of 56.4 (95% CI: 22.8-140.0) (*cf*. Table 2). Conversely, for a joint assay result below S_2.5 _+ T_1000 _the LR^- ^estimate was 0.02 (95% CI: 0.01-0.08), indicating enhanced discrimination against a diagnosis of sCJD when both assay results fall below their individually optimal thresholds (*cf*. Table 1).

**Table 3:** Joint performance characteristics for tau, S100B and 14-3-3

Marker combination	**Bivariate threshold**^**a**^	**LR**^**+ b, c**^	95% CI	**LR**^**- b, d**^	95% CI
Tau + S100B	T_976 _+ S_2.5_^c^	18.0	12.9-25.0	0.02	0.01-0.09
	T_3000 _+ S_2.5_	30.3	18.3-50.3	0.06	0.03-0.13
	T_5000 _+ S_2.5_	35.5	19.2-65.7	0.06	0.03-0.14
	T_1000 _+ S_5.0_	25.1	13.3-47.5	0.12	0.07-0.20
	T_3000 _+ S_5.0_	51.2	20.6-127.5	0.30	0.22-0.40
	T_5000 _+ S_5.0_	57.3	20.7-158.5	0.39	0.31-0.49
Tau + S100B + 14-3-3	T_976 _+ S_2.5_^d^	18.6	13.1-26.3	0.03	0.01-0.10
	T_3000 _+ S_2.5_	29.9	18.0-49.6	0.05	0.02-0.13
	T_5000 _+ S_2.5_	34.9	18.9-64.6	0.05	0.02-0.13
	T_1000 _+ S_5.0_	29.2	14.5-58.7	0.06	0.03-0.14
	T_3000 _+ S_5.0_	49.9	20.0-124.3	0.11	0.06-0.20
	T_5000 _+ S_5.0_	55.6	20.1-154.1	0.11	0.06-0.20

^a ^Bivariate thresholds are defined by combinations of individual thresholds in units of pg/mL (tau) and ng/mL (S100B), as indicated by subscripts.

^b ^LR^+ ^and LR^- ^are positive and negative likelihood ratios, respectively. Values are estimates of joint diagnostic likelihood ratios for CSF tau, S100B and 14-3-3 proteins at illustrative bivariate cutoff thresholds.

^c ^To estimate joint LR^+ ^and LR^- ^values for tau and S100B, the jointly positive patient subgroup was defined as having both tau and S100B results above the chosen pair of thresholds, and the jointly negative patient subgroup was defined as having both tau and S100B results below the chosen pair of thresholds.

^d ^To estimate joint LR^+ ^and LR^- ^values for tau + S100B + 14-3-3, jointly positive and negative subgroups for tau and S100B were defined as in note c, and combined with positive and negative 14-3-3 assay results respectively.

To assess the potential added value of 14-3-3, we also compared AUC estimates for trivariate risk scores for diagnosis of sCJD derived by logistic regression using all three markers as independent variables, to AUC estimates for the bivariate risk estimates derived as above for Tau and S100B. The estimate of AUC for the trivariate risk score was 0.94 (95% CI: 0.93-0.96), significantly smaller than that for the bivariate risk score (P = 0.0297, DeLong test), indicating that if anything, the inclusion of 14-3-3 reduces discriminatory power for a diagnosis of sCJD. Lastly, we calculated LR^+ ^and LR^- ^values jointly for all three markers using the approach based on discrete illustrative cutoff thresholds, and compared them with those obtained for tau and S100B. Based on the very similar LR estimates their broadly overlapping confidence intervals, again we did not observe any evidence for diagnostic value of 14-3-3 beyond that available from tau and S100B (Table 3).

### Post-test probabilities and examples of application

**Figure 4:**
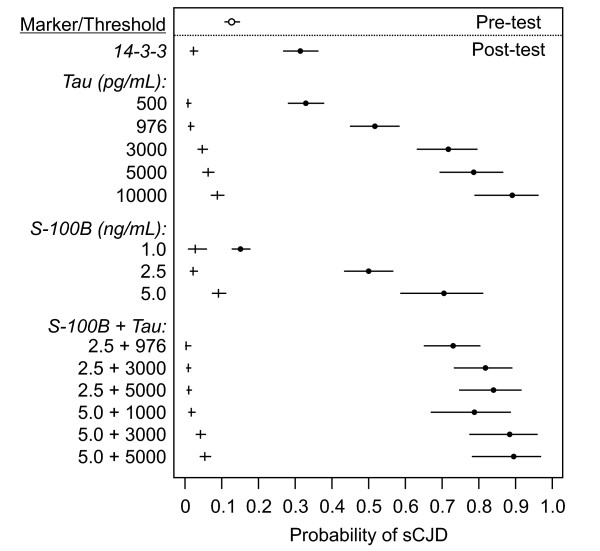
**Post-test probabilities for diagnosis of sCJD**. Point estimates (closed circles for a positive test result; vertical bars for a negative test result) and 95% credible intervals (horizontal bars) of post-test probabilities for a diagnosis of sCJD are plotted for various CSF markers and cutoff thresholds. For each estimate, Bayes' theorem was applied to CSF marker assay results, and 95% credible intervals were estimated by modeling on a beta distribution using the software Post_Test_Probabilities. Pre-test probability of sCJD (based on sCJD prevalence in the overall study population) and 95% confidence interval are indicated by the open circle and the horizontal bar. Cutoff thresholds used are listed on the left.

To explore the diagnostic implications of marker performance characteristics, we used a Bayesian modeling approach [25] to provide point estimates and 95% credible intervals for post-test probability (PTP) of a diagnosis of sCJD given either a positive (PTP^+^) or negative (PTP^-^) assay result (note that PTP^+ ^is logically equivalent to positive predictive value, PV^+^, and PTP^- ^is the complement of negative predictive value, 1 - PV^-^). With optimal cutoff thresholds and a pre-test probability equivalent to the overall prevalence of sCJD in the study population, 0.127 (95% CI: 0.107-0.150), diagnostic probabilities for sCJD were clearly less strongly modified by a positive 14-3-3 result (PTP^+ ^= 0.31, 95% credible interval: 0.27-0.36) than by a positive result for either of the other two markers (PTP^+ ^= 0.52, 95% credible interval: 0.45-0.58 for T_976_; PTP^+ ^= 0.50, 95% credible interval: 0.43-0.57 for S_2.5_) (Figure 4).

The importance of taking into account the quantitative level of the assay result is illustrated, for example with PTP^+ ^= 0.89 (95% credible interval: 0.79-0.96) for T_10,000 _compared with PTP^+ ^= 0.52 (95% credible interval: 0.45-0.58) for T_976 _(Figure 4). The added power afforded by combining tau and S100B is also evident, for example by comparing the PTP^+ ^estimate at the S_2.5 _+ T_976 _bivariate threshold (0.73, 95% credible interval: 0.65-0.80) with those at the identical individual thresholds for S100B (0.50, 95% credible interval: 0.43-0.57 at S_2.5_) and tau (0.52, 95% credible interval: 0.45-0.58 at T_976_). PTP^- ^values were very low (< 0.1) for all markers and marker combinations, partly reflecting the relatively low overall prevalence (pre-test probability) of sCJD in this study population. However, PTP^- ^values for some test results were low enough to indicate that sCJD may effectively be ruled out by some results; *e.g*., below the S_2.5 _+ Tau_976 _bivariate threshold, PTP^- ^= 0.003 (95% credible interval: 0.000-0.009).

For additional perspective on the influence of pre-test probability, we noted that the single most common etiologically defined subset of diagnoses among non-CJD patients consisted of non-CJD neurodegenerative diseases, including Alzheimer's disease (n = 93), Amyotrophic Lateral Sclerosis (n = 3), corticobasal degeneration (n = 5), frontal/frontotemporal dementia (n = 34), Lewy body disease (n = 25), multiple system atrophy (n = 7), olivopontocerebellar atrophy (3), Parkinson's disease (n = 7), Pick's disease (n = 1), primary progressive aphasia (1), progressive supranuclear palsy (n = 6), and other neurodegenerative conditions (n = 6). Restricting attention to this subset of non-CJD patients and applying logic similar to that described above, pre-test probability was 0.36 (95% CI: 0.31-0.42), and at an optimal threshold of T_976_, LR^+ ^for tau became 10.0 (95% CI: 6.5-15.5) with a PTP^+ ^of 0.85 (95% credible interval: 0.79-0.91).

## Discussion

We conducted a large, single-center prospective study of the performance characteristics of CSF 14-3-3, tau and S100B in a clinical population with low average pre-test probability of sCJD (~13%). A number of our findings are relevant to the needs of the practising clinician who may need to make diagnostic decisions in a range of clinical contexts with varying amounts of pre-existing information, and we discuss some of these implications below. Methodologically speaking however, we first note that although sensitivity, specificity, and predictive values are convenient summary statistics to understand the accuracies of diagnostic tests, a more general, versatile and practical approach employs likelihood ratios (LRs) as conversion factors between pre- and post-test odds, which can easily be converted in turn to diagnostic probabilities [20,21]. LRs offer a number of advantages, as they (i) make use of all of the information in a 2 × 2 table; (ii) are less dependent than predictive values on disease prevalence; (iii) enable sequential modification of diagnostic probabilities for individual patients in light of accruing information; and (iv) support the more complete extraction of diagnostic information from quantitative test data by calculating multi-level (interval) LRs [21,28]. They can also serve as convenient generic indices for performance comparisons among diagnostic tests, even those with fundamentally different principles or formats, with LR^+ ^values in the ranges 1-2, 2-5, 5-10, and > 10 representing non-useful, low, moderate and high diagnostic power respectively; for LR^- ^values the corresponding ranges are 0.5-1, 0.2-0.5, 0.1-0.2, and < 0.1 [21].

A number of groups have studied CSF 14-3-3, tau and S100B in sCJD patients and non-CJD controls [13-15,29-36], and some use has been made of LRs [34]. However, to our knowledge this is the first use of LRs to estimate and compare the power of these markers to screen for sCJD in a large, low-prevalence patient population. Our estimates indicated clearly that a positive result for 14-3-3, with its LR^+ ^estimate of 3.1 (95% CI: 2.8-3.6), offers low diagnostic power *versus *tau and S100B with their LR^+ ^estimates of 7.4 (95% CI: 6.9-7.8) and 6.6 (95% CI: 6.1-7.1), respectively. This result is attributable to the lower specificity (0.72) of a positive 14-3-3 test result in our patient population. A similar estimate of lower specificity (0.74) for 14-3-3 was also reported recently in a clinical population with ~4.5-fold higher pre-test probability (*i.e*., prevalence) of sCJD (~59%) [22]. Noting this, and because our estimates of sensitivity for tau and S100B (0.91 and 0.87, respectively) were comparable to those of 14-3-3 but specificities of these two markers (0.88 and 0.87, respectively) were significantly higher in the same patient population, we interpret the observed performance disparities to be more likely due to inherent differences among the markers than to study-specific patient selection, technical factors or choice of scoring thresholds.

We also presented quantitative evidence that tau and S100B each show moderate power to modify diagnostic probabilities of sCJD at their respective optimal cutoff thresholds, and high power when the quantitative assay result is taken into account with the use of interval likelihood ratios calculated at different thresholds. Thus, the extremely high CSF tau concentrations (> 10,000 pg/mL) observed in 34% (41/120) of the sCJD patients tested yielded an interval LR of 56.4, which converts a pre-test sCJD probability of 0.13 to a post-test probability of 0.89. Conversely, very low CSF tau concentrations (< 500 pg/mL) corresponded to an interval LR of 0.06, which converts the same pre-test probability to a post-test probability of 0.01. Regarding the choice of threshold value, we found that in our patient population total diagnostic accuracy as defined by a maximized Youden Index was robustly optimal at values of 976 pg/mL for tau, and 2.5 ng/mL for S100B. Both of these values differ significantly from the widely used consensus thresholds of 1300 pg/mL and 4.2 ng/mL for tau and S100B respectively using the same ELISA kits. However, selection of optima that would be generally applicable among laboratories requires further study.

Strikingly, tau and S100B values jointly above their respective optimal thresholds yielded a joint LR^+ ^of 18.0, significantly higher than achieved by either individual marker at the same thresholds. This effect may reflect the combined severity of distinct underlying pathogenetic processes in sCJD, as accumulation of CSF tau and 14-3-3 proteins is believed to indicate rapid neuronal death [18], while S100B is a largely extraneuronal protein actively secreted from glial cells, suggesting it is primarily a marker of astrogliosis [37]. Point estimates of LR^- ^were also lowered by combining tau and S100B, decreasing from 0.10 and 0.15 for tau and S100B respectively at their individual optimal thresholds to 0.03 at the corresponding bivariate threshold. Similar effects were observed in another recent study [22], although these were not quantified in terms of LRs.

All studies of diagnostic test performance are potentially subject to limitations of design or execution that can lead to imprecision or bias, and thus to an inability to interpret or generalize results [38]. We believe that our large, prospective, sequential, autopsy-based design reduced many of these potential effects, but two specific questions merit discussion. One of these concerns accuracy of case classification. For example, for 55 of our 127 patients with autopsy-confirmed prion disease, despite a CJD-like clinicopathological phenotype and no family history of a similar disease, DNA sequencing information was not available. Conceivably, some of these may have been cases of genetic prion disease rather than sCJD. However, we note that 3 of 77 genetically analyzed cases with a CJD phenotype proved to carry a *PRNP *mutation (E200K in each case); application of the resulting estimate of gCJD prevalence in the study population [3/77 = 0.039 (95% CI: 0.013-0.108)] to the 55 cases without genetic information yields an expected number of unrecognized gCJD cases of 2.1 (95% CI: 0.7-5.9), or < 5% (5.9/127) of the sCJD group. Noting also that diagnostic sensitivity and specificity of CSF 14-3-3, tau and S100B in gCJD are similar to those seen in sCJD [39], we believe that this residual uncertainty is unlikely to detract significantly from our main conclusions. Similarly, the low autopsy rate in our non-sCJD group suggests that some of these 873 cases may indeed have had prion disease. However, we also expect this proportion to be small. More specifically, during the 6 calendar years (2004-2009) overlapping the current study interval for which surveillance data are complete, the CJDSS reported annual sCJD mortality rates in Canada of 1.32, 1.30, 1.20, 0.96, 1.21 and 1.36 per million, respectively (mean, 1.23), suggesting a low, albeit undetermined, number of undetected sCJD cases.

A second potential issue has to do with the limited control that a reference laboratory such as ours has over pre-analytic factors related to sample collection, storage and handling that can in principle affect analytic results. Previous studies have suggested that CSF 14-3-3 and tau proteins are unusually stable in CSF, yielding highly comparable results with the same methods we have employed when samples were subjected to ambient temperatures and/or repeated freeze-thaw cycles [29,40]. Similar methodological studies on the stability of S100B in CSF appear to be lacking, but our estimates of diagnostic sensitivity in our patient population (ca. 90% for all three of the studied markers) suggests that losses of sample reactivity caused by suboptimal pre-analytic conditions did not have a large deleterious effect on the interpretability of our study. With this said however, it is conceivable that suboptimal pre-analytic sample handling may have had quantitative effects in some cases, perhaps explaining some false-negative results or even lowering the estimated optimum cutoff thresholds. Future studies should address these questions.

Lastly, if characteristics of a study population do not adequately reflect those of the patient population to which the clinician wishes to apply the resulting information, it can be difficult to generalize to clinical practice - an effect sometimes called "spectrum bias" [41], or simply "spectrum effect" [42]. Because we studied a heterogeneous population of patients sharing a broad common rationale for CSF testing (*i.e*., suspicion of sCJD), we suggest that our overall results, which represent a weighted average of test performance characteristics for all constituent patient subgroups [42], could prove useful over a broad range of clinical situations. However, we have provided one illustrative example of how the clinician, who can sometimes place the suspected sCJD patient into a particular well-represented subgroup - for example according to membership in an etiologically defined disease category (*e.g*., neurodegenerative disease) - and thus refine pre-test probability, can further enhance the diagnostic power of CSF markers. Using this subgroup criterion with tau protein as the example, we demonstrated how, using nearly identical test cutoff thresholds, PTP^+ ^values rose from 0.52 (95% credible interval: 0.45-0.58) to 0.85 (95% credible interval: 0.79-0.91) for patients judged to have neurodegenerative dementia. Given that clinical examination and diagnostic investigations commonly undertaken for subacute encephalopathies should often enable the placement of a particular patient into such a subgroup [8], this type of illustrative example may prove relevant to clinical practice by helping to better define the meaning of "appropriate clinical context" in relation to use of CSF markers to diagnose sCJD.

As we found that 14-3-3 performed least well among the 3 individual markers studied and that the combination of tau and S100B yielded as much information as all 3 markers combined, focusing on tau and S100B among existing CSF markers may be sufficient for most clinical investigations of sCJD. It may also be appropriate to consider formally incorporating tau and S100B into enhanced WHO surveillance case definitions for sCJD [3]. Apart from diagnostic power, another important criterion of marker utility is availability of a suitable technical format. This is particularly relevant for 14-3-3 proteins, which continue to be assayed using immunoblot methods [18,43] that are inherently difficult to control, optimize and standardize in comparison with ELISA. Although ELISA-format 14-3-3 immunoassays with demonstrated diagnostic utility have been developed [32,44-46], these have not yet seen widespread use or commercial distribution.

## Conclusions

In summary, while acknowledging that CSF protein marker testing in the diagnostic investigation of sCJD should always be carefully linked to clinical context, our key finding is that quantitative CSF tau and S100B assays, particularly in combination, have significant value even in clinical settings where the pre-test probability of sCJD is relatively low, and may be an optimal choice for clinical investigations of sCJD, perhaps with prioritization of tau. It may also be timely to consider formally incorporating these markers into sCJD surveillance case definitions. True *ante mortem *laboratory diagnosis of human prion diseases may eventually be achieved with new approaches based for example on PrP^Sc ^[10,11,47], other markers suggested by CJD pathobiology [48] or discovered by systematic screening [49], or perhaps a combination of these. In the interim however, optimized application of known diagnostic markers will require judicious quantitative assessment of their performance in realistic clinical settings.

## Competing interests

MBC, GHJ, EO, DLG, TC, BCKC and ZW declare that they have no competing interests. NRC receives grant funding from PrioNet Canada and the Canadian Institutes of Health Research; serves as a Board Member and scientific consultant for Amorfix Life Sciences Ltd., and holds stocks and stock options in the latter.

## Authors' contributions

All authors read and approved the final manuscript. MBC: conceived the study, supervised the collection of CSF and genetic data, analyzed the data, and drafted the manuscript. GHJ: performed neuropathology analyses, supervised the collection of clinical data, reviewed the data, and reviewed and co-wrote the manuscript. DLG: collected, validated and collated CSF and genetic data, and reviewed the manuscript. EO, TC: collected, validated and organized clinical data, and reviewed the manuscript. BCKC, ZW: reviewed and validated the statistical analyses, and reviewed and co-wrote the manuscript. NRC: established the network of collaborating Canadian clinicians submitting CSF specimens for 14-3-3 testing, and reviewed and co-wrote the manuscript.

## Pre-publication history

The pre-publication history for this paper can be accessed here:

http://www.biomedcentral.com/1471-2377/11/133/prepub

## Supplementary Material

Additional file 1 **Table S1. Specific non-sCJD diagnoses**. The table lists the names of specific alternate final diagnoses reached for 543 patients initially suspected to have prion disease, and numbers of cases for each diagnosis. Click here for file
