# Supplementary material for: Diagnostic accuracy of cerebrospinal fluid protein markers for sporadic Creutzfeldt-Jakob disease in Canada: a 6-year prospective study
Source: BMC Neurol. 2011 Oct 27;11:133. doi: 10.1186/1471-2377-11-133 (PMC3216246; doi:10.1186/1471-2377-11-133)
Supplement: Additional file 1 — Table S1. Specific non-sCJD diagnoses. The table lists the names of specific alternate final diagnoses reached for 543 patients initially suspected to have prion disease, and numbers of cases for each diagnosis. [file 1471-2377-11-133-S1.DOC]

**Table S1: Specific non-sCJD diagnoses.** The table lists the names of specific alternate final diagnoses reached for 543 patients initially suspected to have prion disease, and numbers of cases for each diagnosis.

| **Diagnosis** | **Cases** | **Diagnosis** | **Cases** | **Diagnosis** | **Cases** |
| --- | --- | --- | --- | --- | --- |
| Acidosis | 1 | HIV encephalopathy | 8 | Porphyria | 1 |
| Acute disseminated encephalomyelopathy | 1 | Hydrocephalus | 8 | Primary biliary cirrhosis | 1 |
| Acute gliomatosis | 1 | Hypertension | 3 | Primary progressive aphasia | 1 |
| Alcoholic encephalopathy | 6 | Hypothyroidism | 2 | Progressive multifocal leukoencephalopathy | 1 |
| Alzheimer’s disease | 93 | Infectious process NOS | 3 | Progressive supranuclear palsy | 6 |
| Amyotrophic lateral sclerosis | 3 | Ischaemia | 2 | Psychosis | 3 |
| Anoxia/hypoxia | 7 | Korsakoff syndrome | 3 | Psychosomatic myoclonus | 1 |
| Astrocytoma | 1 | Leptomeningeal carcinomatosis | 1 | Rasmussen encephalopathy | 1 |
| Attention deficit disorder | 1 | Leukoencephalopathy NOS | 2 | Renal failure | 1 |
| Bell’s palsy | 1 | Lewy Body disease | 25 | Rhomboencephalitis | 1 |
| Bipolar disorder | 1 | Limbic encephalitis | 4 | Schizophrenia | 3 |
| CADASIL | 1 | Lithium toxicity | 1 | Seizure disorder NOS | 4 |
| Cancer NOS | 13 | Lupus | 1 | Septicemia | 1 |
| Carcinomatous meningitis | 2 | Lyme disease | 1 | Shulman’s syndrome | 1 |
| Cerebellar/spinocerebellar syndrome NOS | 7 | Lymphoma | 17 | Steinert’s disease | 1 |
| Cervical-spinal stenosis | 1 | Meningioencephalitis NOS | 1 | Streptococcal meningitis | 1 |
| Corticobasal degeneration | 5 | Meningioma | 1 | Stroke/CVA | 35 |
| Cushing’s disease | 1 | Meningitis NOS | 1 | Subacute sclerosing panencephalitis | 1 |
| Demyelinating disease NOS | 1 | Microvascular disease | 3 | Susac syndrome | 1 |
| Depression | 6 | Mitochondrial disease | 1 | Thiamine deficiency | 1 |
| Diabetes | 5 | Motor neuropathy NOS | 1 | Toxic/metabolic disorder NOS | 8 |
| Drug toxicity NOS | 21 | Multiple system atrophy | 7 | Toxic shock syndrome | 1 |
| Encephalitis NOS | 22 | Multiple sclerosis | 9 | Transient myelitis | 1 |
| Environmental toxicity | 2 | Neurodegenerative process NOS | 4 | Transverse myelitis | 3 |
| Epilepsy | 12 | Neuropsychiatric disorder NOS | 6 | Vascular dementia | 8 |
| Folate deficiency | 1 | Neurosarcoidosis | 2 | Vasculitis | 5 |
| Frontal/frontotemporal dementia | 34 | Neurosyphilis | 1 | Viral encephalitis/meningitis NOS | 19 |
| Glioma | 1 | Olivopontocerebellar atrophy | 3 | Von Economo’s Enephalitis Lethargica | 1 |
| Gliomatosis cerebri | 1 | Paraneoplastic disorders | 20 | VZV encephalitis | 1 |
| Guillain-Barré syndrome | 3 | Parkinson’s disease | 7 | Wernicke-Korsakoff syndrome | 1 |
| Hashimoto encephalitis | 7 | Pick’s disease | 1 | West Nile Virus encephalitis | 2 |
| Hepatic encephalopathy | 4 | Pierre Robin syndrome | 1 | Whipple’s disease | 2 |
| Hepatitis C encephalopathy | 1 | Polymyalgia rheumatica | 1 |  |  |
| Herpes encephalitis/meningitis | 6 | Polyneuropathy NOS | 1 |  |  |
